# Breast cancer is associated to impaired glucose/insulin homeostasis in premenopausal obese/overweight patients

**DOI:** 10.18632/oncotarget.20399

**Published:** 2017-08-23

**Authors:** Raúl M. Luque, Laura M. López-Sánchez, Alicia Villa-Osaba, Isabel M. Luque, Ana L. Santos-Romero, Elena M. Yubero-Serrano, María Cara-García, Marina Álvarez-Benito, José López-Mirand a, Manuel D. Gahete, Justo P. Castaño

**Affiliations:** ^1^ Maimonides Institute of Biomedical Research of Cordoba (IMIBIC), Córdoba, Spain; ^2^ Department of Cell Biology, Physiology and Immunology, University of Córdoba, Córdoba, Spain; ^3^ Hospital Universitario Reina Sofia, Córdoba, Spain; ^4^ CIBERobn, Córdoba, Spain; ^5^ ceiA3, Córdoba, Spain; ^6^ Mammary Gland Unit, Hospital Universitario Reina Sofía, Córdoba, Spain; ^7^ Lipids and Atherosclerosis Unit, Hospital Universitario Reina Sofía, Córdoba, Spain

**Keywords:** breast cancer, glucose, insulin, OGTT, obesity

## Abstract

The association between breast cancer (BCa) presence and altered glucose/insulin metabolism is controversial likely due to an inaccurate insulin resistance (IR) assessment and inappropriate stratification of patients by body-mass index (BMI) and menopausal state. 148 women with suspect of sporadic BCa were stratified by BMI and menopause. Fasting levels of glucose, insulin, glycohemoglobin and selected IR-related and tumor-derived markers were measured. Glucose/insulin levels during OGTT were used to calculate insulin resistance/sensitivity indexes. Analysis of 77 BCa-bearing patients and 71 controls showed an association between BCa and IR as demonstrated by impaired glucose/insulin homeostasis (increased fasting- and OGTT-induced glucose levels) and deteriorated IR indexes, which was especially patent in premenopausal women. The association between BCa presence and IR was markedly influenced by BMI, being obese BCa patients significantly more insulin resistant than controls. BCa presence was associated to elevated levels of IR (glucose, triglycerides) and tumor-derived (VEGF) markers, especially in overweight/obese patients. BCa presence is associated to IR in overweight/obese premenopausal but not in premenopausal normal weight or postmenopausal women. Our data support a bidirectional relationship between dysregulated/imbalanced glucose/insulin metabolism and BCa, as tumor- and IR-markers are correlated with the impairment of glucose/insulin metabolism in overweight/obese premenopausal BCa patients.

## INTRODUCTION

Breast cancer (BCa) is the most frequent and the second leading cause of cancer-related death in female population [1]. BCa is conditioned by genetic predisposition and environmental factors, such as obesity [2–5], diabetes [6], or metabolic syndrome [6–8]. All these BCa-associated risk/prognostic factors share several hallmarks, wherein insulin resistance (IR) seems to have particular relevance [9, 10]. Indeed, IR is associated to hyperinsulinemia, hypertension, and impaired glucose tolerance, and, consequently, has been proposed as a putative underlying cause in the relationship of obesity, diabetes, and metabolic syndrome with higher BCa risk [2, 8, 11, 12], prognosis [7, 13] and survival [3, 14]. However, the direct, causative association between IR and BCa is still controversial [15–19] in that, although fasting glucose seems to be a risk factor for BCa [20], data regarding other IR markers (insulin/C-peptide) and their role in BCa development/progression are inconclusive, as some studies reported an association [16, 18], whereas others did not [15, 17, 19]. It is also controversial whether BCa presence is associated to IR and/or dysregulated or imbalanced glucose and insulin metabolism. Indeed, although a recent study reported higher fasting glucose and insulin levels in non-diabetic BCa patients [13], a previous meta-analysis found no alteration of IR markers in BCa patients [21]. In this scenario, it has been suggested that the lack of convincing data regarding a putative association between IR and BCa could be due to the fact that IR has not been appropriately evaluated and/or analyzed. Thus, some authors have proposed that oral glucose tolerance test (OGTT) should be used, for impairments in glucose metabolism are better identified dynamically, in response to a glucose challenge [21, 22]. Additionally, studies analyzing the relationship between IR and BCa could be biased by a lack of correct patient stratification in terms of body-mass index (BMI) and menopausal state, in that both factors are directly associated to IR and BCa [23–25]. Finally, it should be noted that several other IR-related factors are associated to BCa [altered lipids, cytokines, adipokines, insulin-like growth factor I (IGF-I), Tumor necrosis factor alpha (TNF-alpha), pro-angiogenic factors, estrogens, etc.], which could be modulating/masking the putative direct relationship between IR and BCa [12].

Accordingly, in an attempt to unequivocally elucidate the association between BCa and glucose/insulin metabolism, by more accurately assessing IR, and by appropriately stratifying the patients by BMI and menopausal state, we have implemented a prospective study including 148 women with suspect of non-familial, sporadic BCa in whom we measured glucose, insulin, glycohemoglobin, and other IR-related and tumor-derived markers, as well as glucose/insulin levels during the OGTT. Our initial hypothesis was that there is a marked association between BCa presence and alterations in glucose/insulin metabolism, which could be influenced by BMI and menopausal status.

## RESULTS

### Study population

A total of 148 women with suspect of non-familial, sporadic BCa were included in this prospective study (77 BCa patients and 71 controls). Demographic and clinical data are shown in Table 1. Mean age (*p* < 0.001) and postmenopausal status proportion (*p* = 0.001) were higher in BCa group compared to controls, whereas obesity-related parameters (body weight, BMI, waist circumference or cholesterol levels) did not differ (Table 1).

**Table 1:** Demographic and clinical parameters of the cohort

	Control (*n* = 71)	Breast cancer (*n* = 77)	*p*-value
Age (years)	44.0 ± 1.3	53.6 ± 1.2	< **0.001**
Weight (kg)	67.9 ± 1.3	69.2 ± 1.2	0.480
BMI (kg/m^2^)	26.2 ± 0.5	26.9 ± 0.5	0.316
Systolic pressure (mm Hg)	112.7 ± 1.9	127.0 ± 2.6	< **0.001**
Diastolic pressure (mm Hg)	69.7 ± 1.4	75.4 ± 1.8	**0.034**
Waist perimeter (cm)	86.3 ± 1.3	89.1 ± 1.4	0.160
Number of children	1.9 ± 0.09	2.3 ± 0.1	0.124
Age of menarche	12.3 ± 0.2	12.6 ± 0.1	0.241
Age of first childbirth	26.2 ± 0.7	25.0 ± 0.6	0.221
Premenopausal state (%)	77.0	55.0	**0.001**
Postmenopausal state (%)	23.0	45.0	**0.001**
Total cholesterol (mg/dL)	201.2 ± 4.4	210.6 ± 3.9	0.116
HDL cholesterol (mg/dL)	53.6 ± 1.5	53.8 ± 1.4	0.950
LDL cholesterol (mg/dL)	127.5 ± 3.7	134.6 ± 3.6	0.109

Values represent mean ± SEM of each parameter in control (*n* = 71) and breast cancer (*n* = 77) groups. *P*-value indicates differences by Mann-Whitney, Student *t*-test and/or Fisher exact test. *p* < 0.05 was considered significant.

### BCa patients exhibited impaired glucose/insulin metabolism

**Figure 1:**
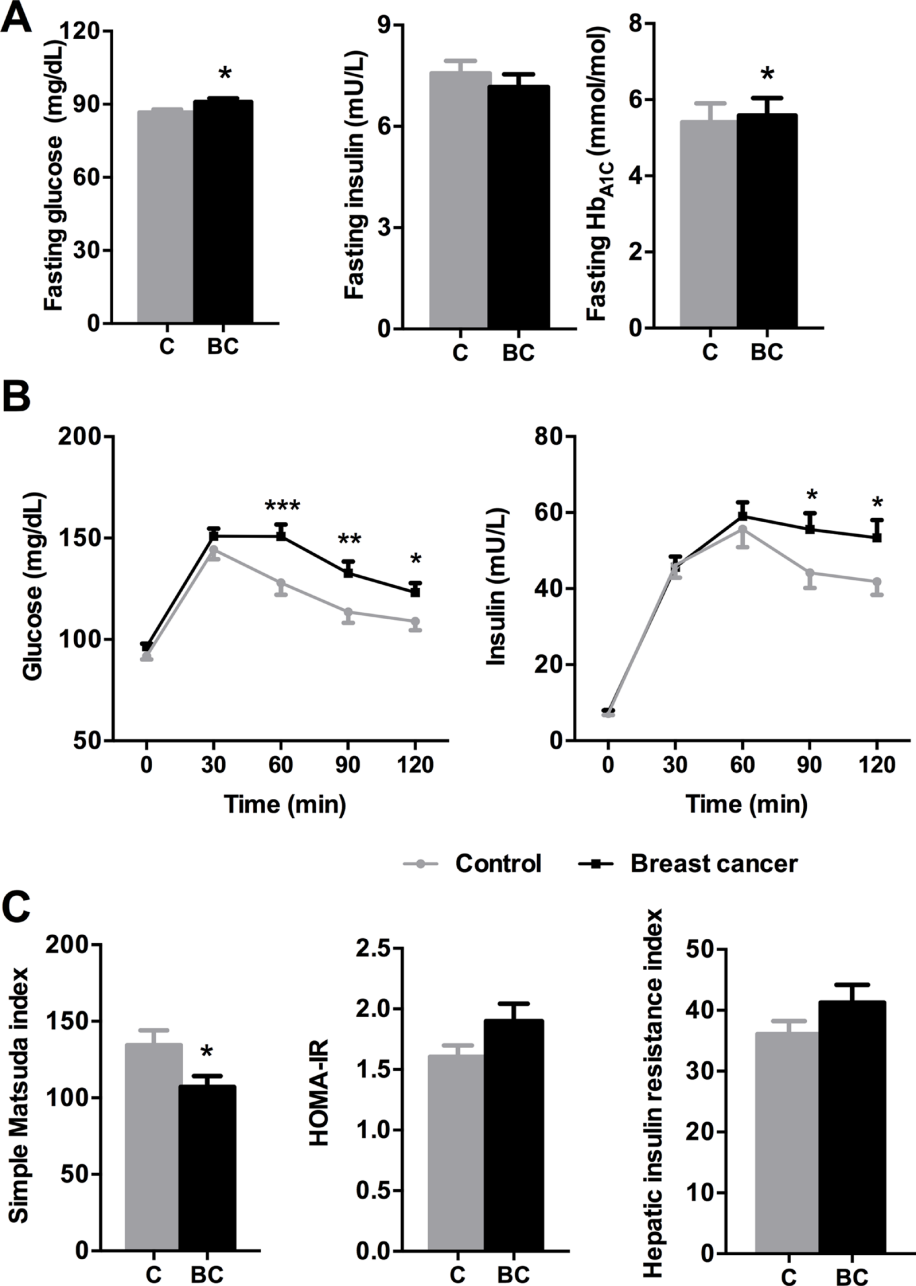
Glucose/insulin metabolism parameters in control and breast cancer patients (**A**) Fasting glucose, insulin and glycohemoglobin levels were analyzed. Asterisks (*p < 0.05) indicate differences by Mann-Whitney U/Student *t*-test. (**B**) Glucose and insulin levels during the OGTT. Asterisks (*p < 0.05; **p < 0.01; ***p < 0.001) indicate significant differences between groups by Fisher's LSD test. (**C**) Simple Matsuda index, HOMA-IR and Hepatic insulin resistance index. Asterisks (*p < 0.05) indicate differences by Student *t*-test. Values represent means ± SEM of control (*n* = 71) and breast cancer (*n* = 77) groups. C means control subjects and BC means breast cancer patients.

BCa patients presented significantly higher fasting levels of glucose and glycohemoglobin compared to controls, without changes in fasting insulin (Figure 1A). Additionally, OGTT was drastically impaired in BCa patients (BCa presence effect: *p* = 0.014) in that glucose levels remained more elevated in BCa patients at 60, 90 and 120 min after glucose load, with no changes during the first phase (0–30 min), suggesting peripheral IR (Figure 1B). Similarly, insulin levels remained more elevated in BCa patients during OGTT at 90 and 120 min (Figure 1B). Accordingly, Simple Matsuda Index (SMI) was lower in BCa individuals; while the Homeostatic Model Assessment of Insulin Resistance (HOMA-IR) and the Hepatic Insulin Resistance Index (HIRI) of BCa patients were numerically but not significantly elevated (*p* = 0.392 and *p* = 0.472, respectively) compared to controls (Figure 1C). Remarkably, tumor type or receptor status had no significant effect on these associations (data not shown).

**Figure 2:**
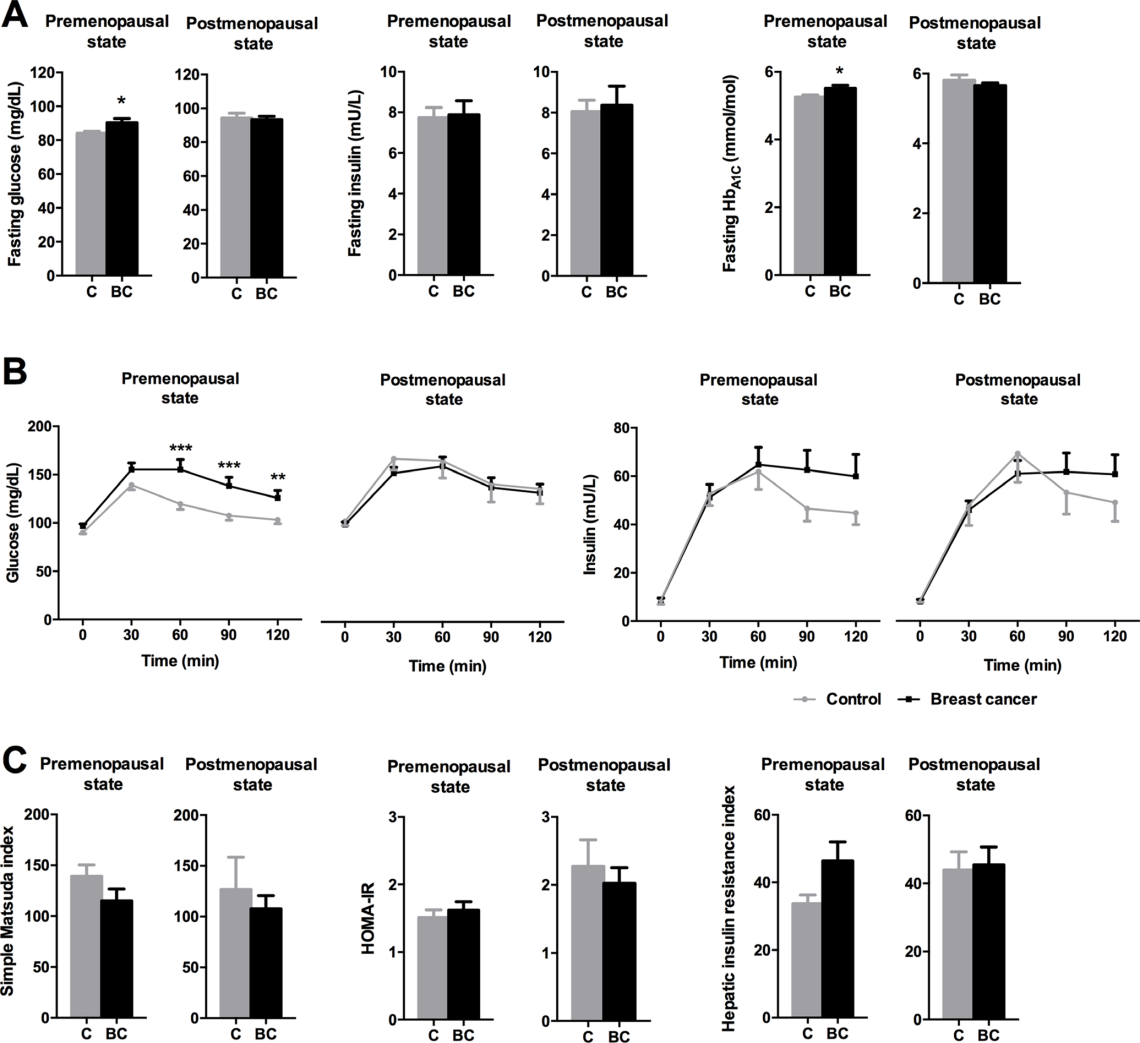
Impact of menopausal status on glucose/insulin metabolism parameters in control and breast cancer patients (**A**) Fasting glucose, insulin and glycohemoglobin levels were analyzed. Asterisks (*p < 0.05) indicate differences by Student *t*-test. (**B**) Glucose and insulin levels during the OGTT. Asterisks (**p < 0.01; ***p < 0.001) indicate significant differences by Fisher's LSD test. (**C**) Simple Matsuda index, HOMA-IR and Hepatic insulin resistance index. Values represent means ± SEM. C means control subjects and BC means breast cancer patients.

Subsequently, as menopause could be associated with BCa, obesity, and glucose/insulin metabolism, we subdivided both groups by menopausal state (Supplementary Table 1). This analysis revealed that premenopausal BCa patients had higher glucose and glycohemoglobin levels and impaired OGTTs compared to premenopausal controls (Figure 2); oppositely, no differences were found between postmenopausal patients. In addition, premenopausal BCa patients presented a non-significant trend for higher HIRI (*p* = 0.08; Figure 2). Of note, postmenopausal controls exhibited increased glucose and glycohemoglobin levels, and impaired OGTTs compared to premenopausal controls (Supplementary Figure 1).

As age, a risk factor for BCa and obesity incidence, was higher in BCa patients (Table 1 and Supplementary Table 1), general linear models for repeated measurements were implemented to clarify its putative influence on the parameters evaluated. Considering the whole cohort, this analysis confirmed a significant effect of BCa presence on OGTT response. However, this effect disappeared when age was used as covariate, indicating an effect of age on OGTT response (Supplementary Table 2). Furthermore, this model confirmed a significant effect of BCa presence in premenopausal women, which was maintained when corrected by age (Supplementary Table 2). This data was further validated by selecting a subgroup of premenopausal age-matched control and breast cancer patients (average age of 44.0 and 44.4 years, respectively; *p*-value = 0.709 by Student *t*-test) and analyzing OGTT response, which confirmed a significant effect of BCa on OGTT response in premenopausal women when corrected by age (Supplementary Figure 2).

### Glucose/insulin metabolism was altered in obese, but not in normal weight, BCa patients

**Figure 3:**
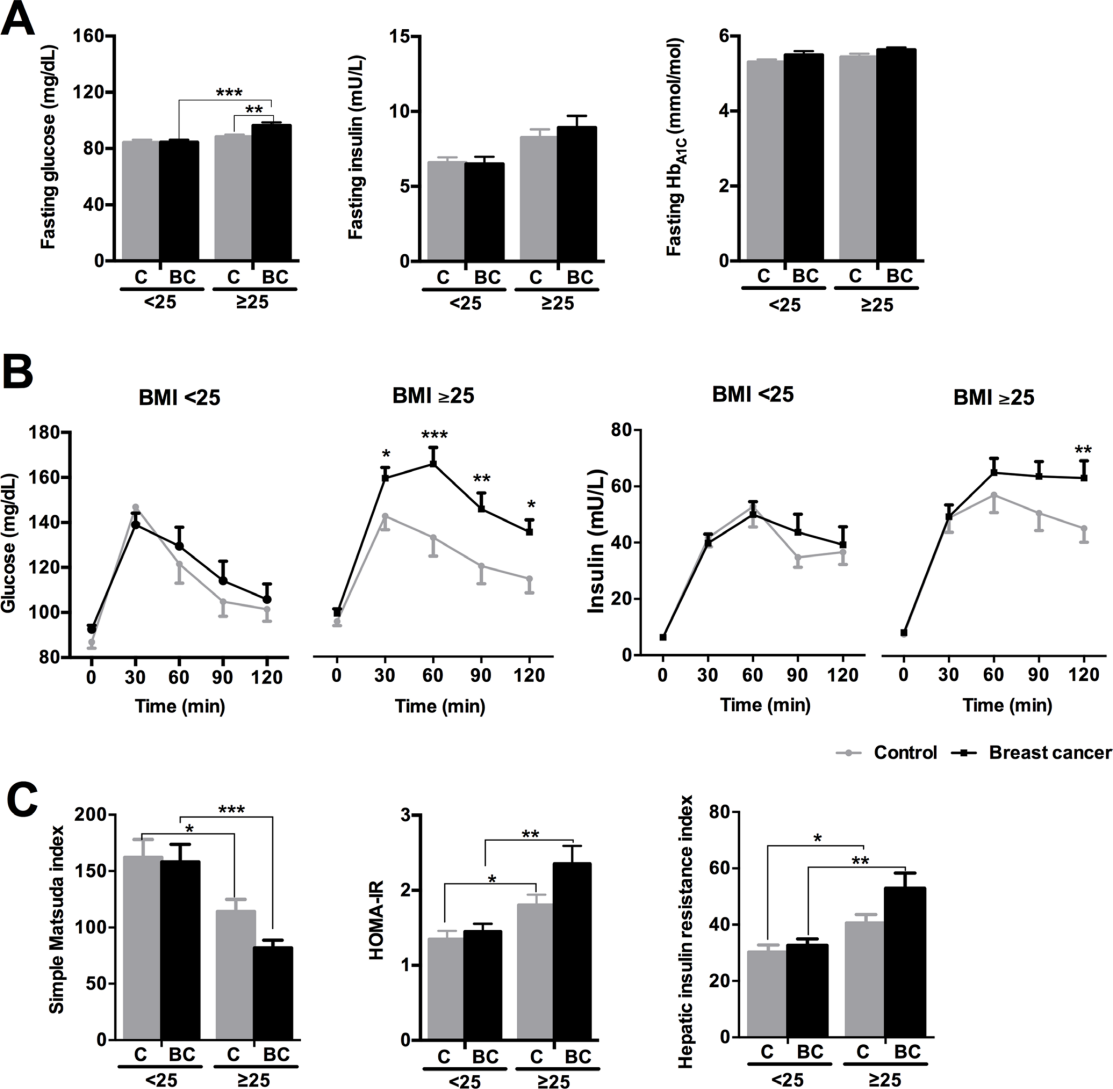
Glucose/insulin metabolism parameters in normal weight (BMI < 25) and overweight/obese (BMI ≥ 25) control and breast cancer patients (**A**) Fasting glucose, insulin and glycohemoglobin levels. (**B**) Glucose and insulin levels during the OGTT. (**C**) Simple Matsuda index, HOMA-IR and Hepatic insulin resistance index. Values represent mean ± SEM of each experimental group (normal weight *n* = 60; overweight *n* = 88). Asterisks (*p < 0.05; **p < 0.01; ***p < 0.001) indicate significant differences by Bonferroni/Mann-Whitney U/Fisher's LSD post-hoc tests. C means control subjects and BC means breast cancer patients.

When normal weight (BMI < 25 kg/m^2^) and overweight/obese (BMI ≥ 25 kg/m^2^) patients were considered separately, a strong association between BCa and impaired glucose/insulin homeostasis was observed in overweight/obese individuals but not in normal weight women (Figure 3). Fasting glucose and insulin levels were affected by body weight, but only fasting glucose levels were influenced by BCa presence, being significantly more elevated in overweight/obese (BMI ≥ 25) BCa patients than in normal weight BCa patients, and, importantly, than in overweight/obese (BMI ≥ 25) controls (Figure 3A). Furthermore, whereas OGTT was not altered in BCa-bearing normal weight patients compared to normal weight controls (Figure 3B), BCa patients with BMI ≥ 25 showed impaired OGTT compared to obese controls, as glucose levels remained significantly elevated at 30, 60, 90 and 120 min, and insulin levels did not recover after 60 min, reaching a significant difference at 120 min vs. obese controls (Figure 3B). Indeed, IR indexes were clearly influenced by body weight within both, control and BCa groups (Figure 3C); however, despite the overt numerical differences, only non-significant trends were found between overweight/obese BCa patients and controls (Figure 3C).

Remarkably, premenopausal BCa patients had a more pronounced impairment of glucose/insulin metabolism than their controls, especially in patients with BMI ≥ 25 (Supplementary Figure 3). In fact, fasting glucose and glycohemoglobin levels were not altered in BCa normal weight premenopausal women compared to controls, but tended to be higher (glucose; *p* = 0.072) or were significantly elevated (glycohemoglobin; *p* < 0.05) in BCa premenopausal patients with BMI > 25 compared to controls (Supplementary Figure 3A). In contrast, fasting glucose was significantly lower in BCa normal weight postmenopausal patients compared to controls (Supplementary Figure 3A). Accordingly, overall glucose levels were not significantly altered during the OGTT by BCa presence in premenopausal normal weight subjects (*p* = 0.153), despite slightly elevated glucose levels at 60 and 90 min in BCa patients vs. controls (Supplementary Figure 3B). Conversely, BCa presence was clearly associated to impaired OGTT in premenopausal BCa patients with BMI ≥ 25 compared to premenopausal controls, with higher glucose levels at 30, 60, 90 and 120 min (Supplementary Figure 3B). In contrast, no body weight-related differences were found for glucose in OGTT between BCa vs. control in postmenopausal women (Supplementary Figure 3B). Similarly, only premenopausal BCa patients with BMI ≥ 25 showed a non-significant trend for elevated insulin levels after 120 min (*p* = 0.083) (Supplementary Figure 3B). Furthermore, SMI tended to be reduced in BCa compared to controls in premenopausal women (*p* = 0.053, two-way ANOVA), but not in postmenopausal subjects. Interestingly, the differences within these groups seemed to be especially drastic in women with BMI > 25 (Supplementary Figure 3C).

### The severity of glucose/insulin metabolism impairment in BCa patients is directly associated to BMI

**Figure 4:**
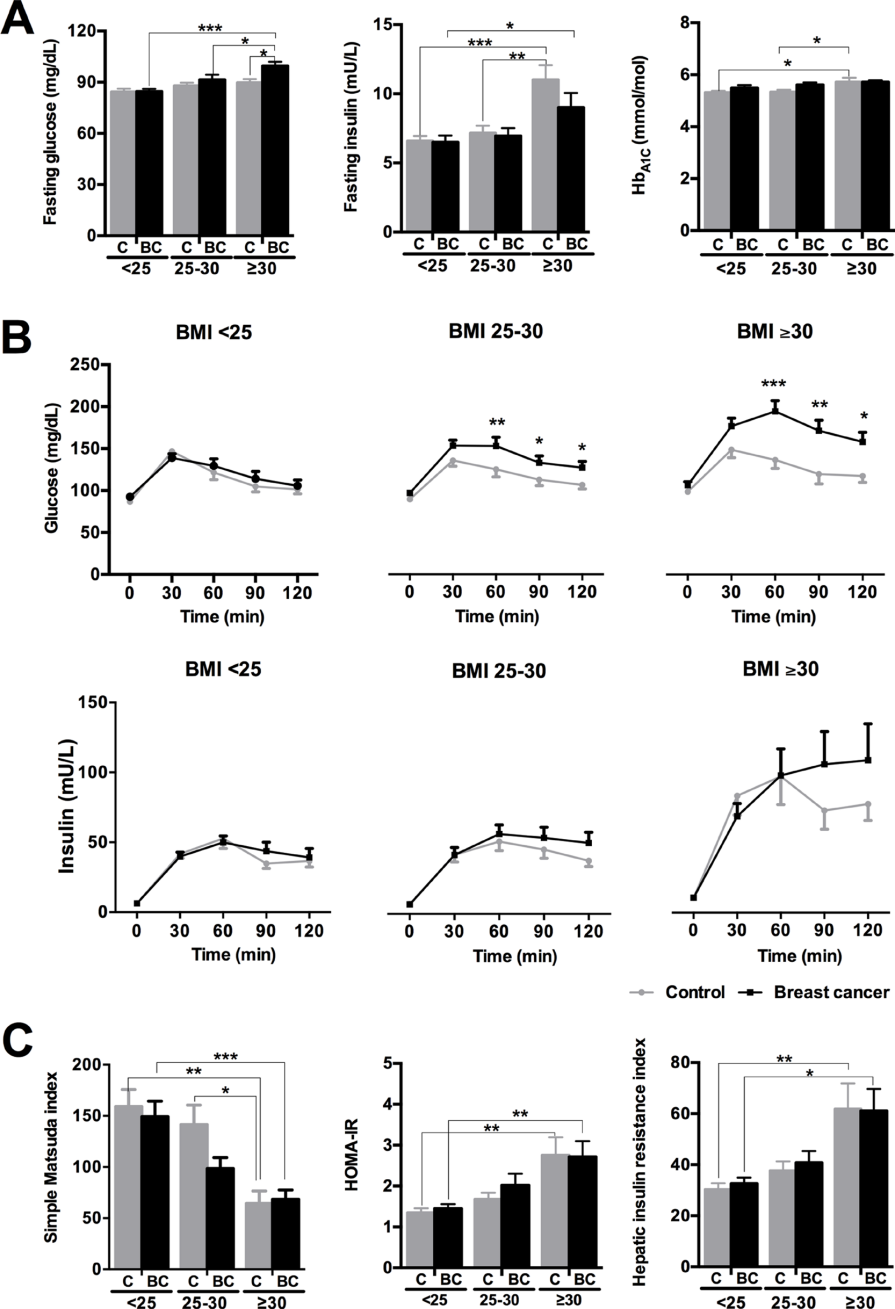
Glucose/insulin metabolism parameters in normal weight (BMI < 25), overweight (BMI ≥ 25 < 30) and obese (BMI ≥ 30) control and breast cancer patients (**A**) Fasting glucose, insulin and glycohemoglobin levels. (**B**) Glucose and insulin levels during the OGTT. (**C**) Simple Matsuda index, HOMA-IR and Hepatic insulin resistance index. Values represent mean ± SEM of each experimental group (normal weight *n* = 60; overweight *n* = 54; obese *n* = 34). Asterisks (*p < 0.05; **p < 0.01; ***p < 0.001) indicate significant differences by Bonferroni/Mann-Whitney U/Fisher's LSD post-hoc tests. C means control subjects and BC means breast cancer patients.

The analysis implemented when BMI ≥ 25 patients were subdivided into overweight (25 ≥ BMI < 30) and obese (BMI ≥ 30) showed that fasting glucose levels were significantly increased in obese BCa patients compared to normal weight and overweight BCa patients, and to obese controls (Figure 4A). Fasting insulin levels were elevated in obese individuals compared to the respective normal weight controls and obese BCa patients (Figure 4A). Interestingly, glycosylated hemoglobin was only elevated in obese controls compared to normal weight and overweight controls (Figure 4A). In addition, glucose levels during the OGTT remained more elevated in BCa obese patients than in BCa overweight patients, compared to their corresponding controls (Figure 4B). Moreover, OGTT curves of obese BCa patients were significantly different to those from normal weight and overweight BCa patients, whereas in controls, OGTT response was only statistically different between normal weight and obese individuals (Supplementary Figure 4). Consistently, insulin levels during the OGTT remained elevated only in BCa obese patients compared to controls (BCa presence effect: *p* = 0.012) (Figure 4B). Finally, IR indexes were clearly different between normal weight and obese individuals, but did not reflect an influence of BCa presence (Figure 4C).

### Determinants of the association between BCa and impaired glucose/insulin metabolism

**Figure 5:**
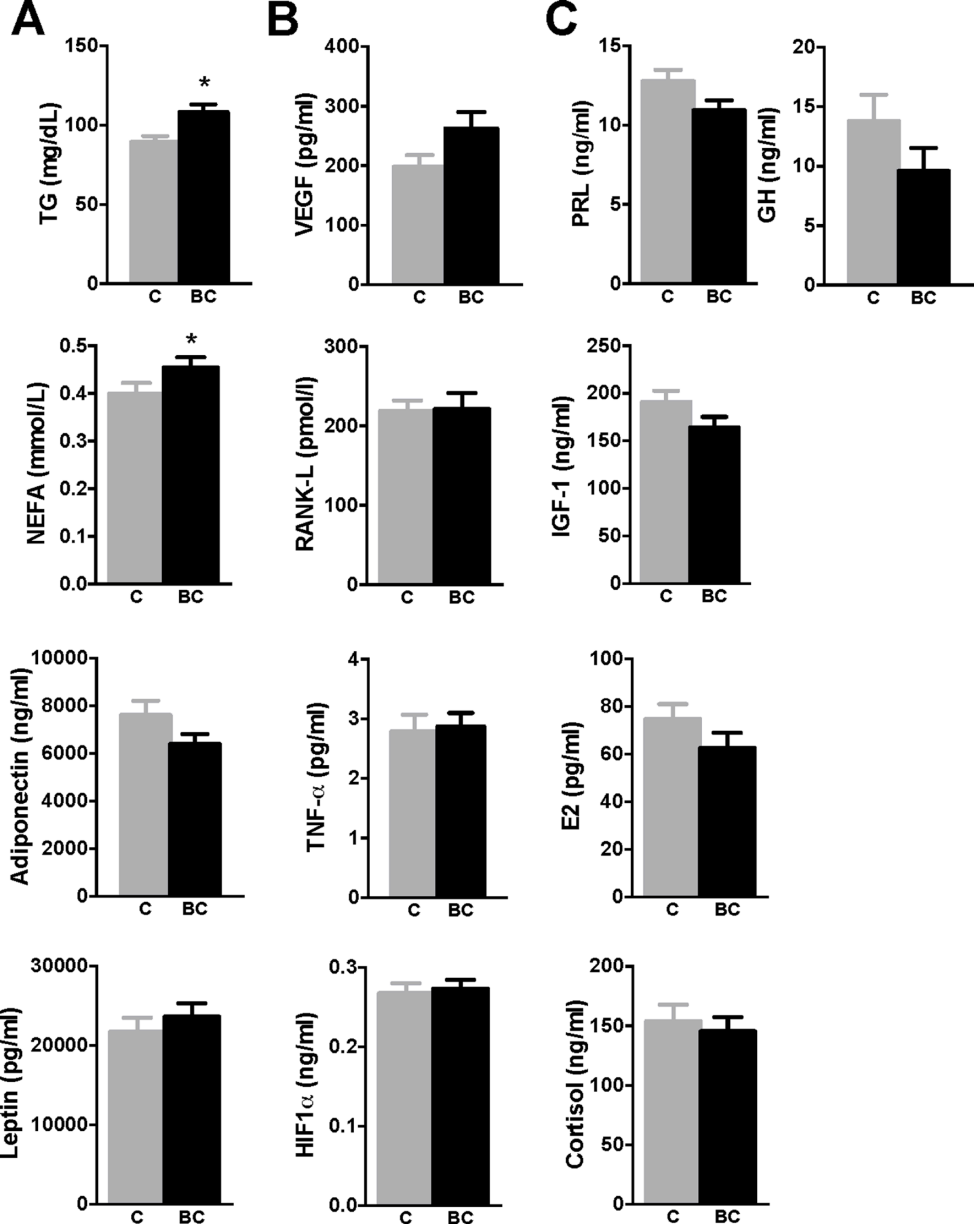
Circulating levels of relevant hormones, factors and molecules in control and breast cancer patients (**A**) Obesity-associated markers (TG, NEFA, adiponectin, leptin), (**B**) tumor-derived factors (VEGF, RANK-L, TNF-α, HIF-1α) and (**C**) other relevant hormones [PRL, IGF-I, estrogens (E2), cortisol, GH] were determined in plasma of cohort individuals by specific ELISAs. Values represent mean ± SEM of each experimental group (control *n* = 71; breast cancer *n* = 77). Asterisks (*p < 0.05) indicate significant differences between groups by Student *t*-test/Mann-Whitney *U* test. C means control subjects and BC means breast cancer patients.

To ascertain the putative causal relationship between presence of BCa and impaired glucose/insulin metabolism, we have determined the circulating levels of obesity- and BCa-associated factors. As shown in Figure 5A, certain obesity-associated markers such as triglycerides (TG) or non-esterified fatty acids (NEFA) were increased in BCa patients compared to controls, while leptin or adiponectin were not significantly altered. Similarly, Vascular Endothelial Growth Factor (VEGF), a tumor-derived factor, tended (*p* = 0.061) to be increased in BCa patients; while other putative tumor-derived factors such as RANK-L, Hypoxia-inducible Factor 1 alpha (HIF1α) or TNF-α were not significantly altered (Figure 5B). Additionally, other hormones [Growth hormone (GH), IGF-I, estrogens, cortisol) were non-significantly altered, except for prolactin, which tended (*p* = 0.057) to be lower in BCa patients. Consistently, when the patients were stratified according to the BMI, similar changes/tendencies were found (Supplementary Figures 5 and 6), confirming that TG, NEFA, VEGF and prolactin were the factors altered by the presence of BCa, specially in overweight and/or obese patients.

## DISCUSSION

Although BCa is strongly influenced by obesity, diabetes and metabolic syndrome [2–4, 6–8, 11], the pathophysiological mechanisms and the molecular and cellular determinants underlying this association are not fully elucidated [26]. Among the players proposed, IR represents a leading candidate, in that it is a major component of metabolic syndrome, diabetes and other metabolic diseases, as well as of several cancer types including BCa. However, the results reported hitherto are controversial [15–19], likely due to the differences in the methods employed to assess IR, and disparities in the intrinsic particularities of the study cohorts, as is the case of BMI or menopausal state. This study was devised to unequivocally unveil the potential association between BCa and IR, by assessing this parameter more accurately, and by stratifying the patients appropriately by BMI and menopausal state. Thereby, this prospective study revealed the existence of a clear association between BCa and IR, which is particularly drastic in premenopausal women and is strongly influenced by BMI.

Indeed, the results presented herein demonstrate, for first time, that premenopausal overweight and obese BCa patients exhibit a significantly impaired OGTT compared to that of BMI- and menopausal state-matched controls, whereas this association is not patent in premenopausal normal weight patients nor in postmenopausal women. Interestingly, previous studies have shown that overweight BCa patients near to the initiation of chemotherapy (*n* = 8) have worsened OGTT compared to age- and BMI-matched controls [22], that OGTT improved after 6 months chemotherapy in ten previously undiagnosed diabetic postmenopausal BCa women [27], and that non-diabetic overweight BCa patients present higher fasting glucose and insulin levels [13]. However, this study provides the first evidence that naive, untreated BCa patients exhibit worsened OGTT compared to matched controls, and that this is predominantly observed in premenopausal women and drastically influenced by BMI. Indeed, normal weight premenopausal BCa patients did not exhibit signs of impaired glucose tolerance or IR compared to normal weight controls, thereby suggesting that, under normal weight conditions, presence of BCa is not associated to worsened glucose/insulin metabolism. Conversely, under conditions of deteriorated glucose management (overweight/obesity), presence of BCa was associated to aggravated IR and impaired glucose homeostasis, which could even worsen the outcome of overweight/obese BCa patients. Importantly, our observation could be related to the fact that the relative risk of mortality is higher in obese BCa patients compared to controls [3, 4, 28, 29]. In contrast to premenopausal women and despite obesity and metabolic syndrome are associated to worst BCa outcome and survival especially in postmenopause [2–4, 11, 28], BCa postmenopausal patients did not present altered glucose/insulin metabolism irrespective of BMI. This observation could be associated to the fact that postmenopausal woman already exhibit impaired glucose/insulin metabolism compared to premenopausal women ([30, 31] and data presented herein). In any case, it should be noted that a limitation of this study is that body fat mass and body fat distribution of the patients, two parameters that could be influencing insulin resistance, were not determined, and, therefore, this issue warrants future investigation

Nevertheless, it is yet uncertain whether dysregulated glucose/insulin homeostasis, particularly in obese women, could cause/enhance BCa development and progression and/or, on the other hand, BCa presence could induce/exacerbate a situation of impaired glucose/insulin metabolism. Likewise, the precise mechanisms and cellular/molecular players underlying this uni- or bidirectional relationship are not fully known. Several studies have provided evidence favoring the notion that a reciprocal influence can occur, although the weight of the data in each direction is not balanced. Specifically, mounting evidence indicate that several obesity-associated factors such as insulin, leptin, adiponectin or triglycerides can contribute to the development and/or progression of several cancer types, including BCa [32, 33]. Likewise, there is also data supporting the contention that the tumor could influence whole body glucose/insulin metabolism through the secretion of certain factors [34–40]. In an attempt to shed some light in this issue, we determined circulating concentrations of selected obesity- and tumor-derived factors. Consistent with previous studies [20], glucose and glycosylated hemoglobin were elevated in BCa patients, especially in those exhibiting a more pronounced impairment of glucose/insulin metabolism (premenopausal patients with higher BMI) compared to controls. However, insulin levels were not significantly altered in the experimental groups, an observation that compares well with previous studies [21], and suggests that insulin is unlikely to be solely responsible for this association. Interestingly, TG and NEFA, other IR-associated markers [41, 42], which are involved in BCa development/progression [43, 44], were elevated in BCa patients compared to BMI-matched controls, thus underscoring the interest in further studying the potential contribution and underlying mechanisms of these factors into BCa. Additionally, from the tumor-associated factors analyzed (VEGF, RANK-L, TNF-alpha and HIF-1alpha) [45–48] that could be putatively associated to the impaired glucose/insulin metabolism [35, 39, 40, 49], only VEGF was found overtly elevated in BCa patients compared to controls. The bidirectional relationship between VEGF and impaired glucose/insulin homeostasis in BCa has not been studied in detail hitherto, apart from some insightful animal studies indicating that VEGF-C overexpression induces weight gain and IR [34]. Our findings suggest that this putative interaction deserves a more careful examination, as the data presented herein provide evidence that a multifactorial causative reciprocal interconnection may underlie the pathological association between IR and BCa in overweight/obese premenopausal women, wherein insulin-resistance associated factors (glucose, TG, FFA) could potentiate BCa development/progression while tumor-derived factors (VEGF) could aggravate IR.

In summary, our results indicate that BCa presence is associated to higher IR in overweight/obese premenopausal woman but not in premenopausal normal weight patients or in postmenopausal woman. Further, our findings suggest a bidirectional relationship between dysregulated glucose/insulin metabolism and BCa, as both, tumor- and IR-related markers are correlated with the severity of glucose/insulin metabolism impairment in overweight/obese premenopausal BCa patients.

## MATERIALS AND METHODS

### Ethics, consent and permissions

The study protocol was approved by the Human Investigation Review Committee of the Reina Sofia University Hospital (HURS, Cordoba, Spain). Patient management followed the guidelines established by the internal “Breast Cancer Patients Protocol”. We had all necessary consents from patients involved in the study, including consent to participate in the study.

### Participants

Between 2011 and 2015, women received in the Mammary Gland Unit at the HURS with suspect of non-familial, sporadic BCa, considered within a group of common risk, image-diagnosed within BIRADS (Breast Imaging Reporting and Data System) grade 4–5 and subjected to core-needle biopsy of the mammary gland were invited to participate in this study. Particularly, 148 women (mean age: 49.3 ± 0.9; range: 21–81) accepted to participate in this prospective study. After the diagnosis (core-needle biopsy), patients were classified as “Control” (*n* = 77) or “Breast Cancer” (*n* = 71).

### Study design

The clinical protocol included: 1) collection of personal data, family history, associated pathologies and menopausal state; 2) evaluation of body mass index, waist perimeter and blood pressure; 3) collection of blood in fasting state; and 4) OGTT performance. According to current laws and legal procedures, all the personal information collected was confidential, and all biological samples and the results obtained were codified. Those patients with family history of BCa or diseases related to glucose metabolism (diabetes) and other chronic illnesses were excluded.

### Blood sampling, OGTT and biochemical analysis

Sessions started between 8:30–9:30 am and all participants were fasted for 8–12 hours (no food or drink except for water). Fasting blood samples were obtained for biochemical analysis. Then, two fasting samples were drawn (times −15 min and 0 min, respectively); immediately after, an oral overload of 75 gr of glucose drink was provided. At 30, 60, 90 and 120 min [50], blood samples were obtained and spread into three tubes (BD Vacutainer, New Jersey, US): EDTAK_3_-treated plasma, oxalate-treated plasma and serum-separating tubes. Plasma and oxalate-treated blood were obtained after centrifugation of 10 min at 3000 rpm (4°C), while serum was obtained by centrifugation of 20 min at 2500 rpm (room temperature). Plasma was frozen at −80°C, while 500 μl of each oxalate-plasma and serum sample were used for glucose (Hexokinase/G-6-PDH technique) and insulin (ARCHITEC i2000SR, Abbott Diagnostic) determinations.

### Determination of insulin sensitivity/resistance indexes

Fasting indexes were calculated using the formula: HOMA-IR = [fasting insulin (mU/L)*fasting glucose (mmol/L)]/22.5 and *Hepatic insulin resistance Index* (HIRI) = fasting glucose*fasting insulin. From OGTT, *Simple Matsuda Index* (an index to evaluate whole body insulin sensitivity) was calculated as: SMI = 10.000/square root of [fasting glucose*fasting insulin]*[glucose at time 120*insulin at time 120].

### Measurements of circulating hormones and metabolites

Fasting blood was used to determine glucose, insulin, glycohemoglobin, lipids (non-esterified fatty acids [NEFA (Wako Chemicals GmbH, Neuss, DE)], total cholesterol [TC], low-density lipoprotein cholesterol [LDL], high density lipoprotein cholesterol [HDL] and triglycerides [TG]). Also, circulating levels of GH, IGF-1, cortisol and PRL (DRG International, Inc., US); leptin, TNF-α, VEGF (Invitrogen, CA, US), adiponectin (R&D Systems, MN, US), RANK-L (BioVendor, CR, US), HIF1α (RayBiotech, GA, US) and estradiol (Life Technologies, CA, US) were measured using commercial ELISA kits. All the information regarding each assay can be accessed at the company website.

### Statistics

To evaluate data variation in OGTT measurements, analysis of variance for repeated measures (2-way ANOVA) was performed, followed by Fisher's correction test for multiple comparisons (the effect of BCa/menopause/BMI and time was determined). To analyze the influence of age in the results derived from the OGTT, a linear regression analysis was carried out. Moreover, Student *t*-test, Mann-Whitney and 2-way ANOVA (followed by Bonferroni's post-hoc test) tests were used, according to the number of experimental groups analyzed and the normality of the data. All data are expressed as mean ± SEM. Differences were considered to be significant when *p* < 0.05. GraphPad Prism 6.0 software (La Jolla, CA) and SPSS 24.0 (IBM) were used for the statistical analysis.

## SUPPLEMENTARY MATERIALS FIGURES AND TABLES


