# Supplementary material for: Breast cancer is associated to impaired glucose/insulin homeostasis in premenopausal obese/overweight patients
Source: Oncotarget. 2017 Aug 23;8(46):81462–74. doi: 10.18632/oncotarget.20399 (PMC5655300; doi:10.18632/oncotarget.20399)
Supplement: Supplementary file 1 [file oncotarget-08-81462-s001.pdf]

Breast cancer is associated to impaired glucose/insulin homeostasis in premenopausal obese/overweight patients

SUPPLEMENTARY MATERIALS

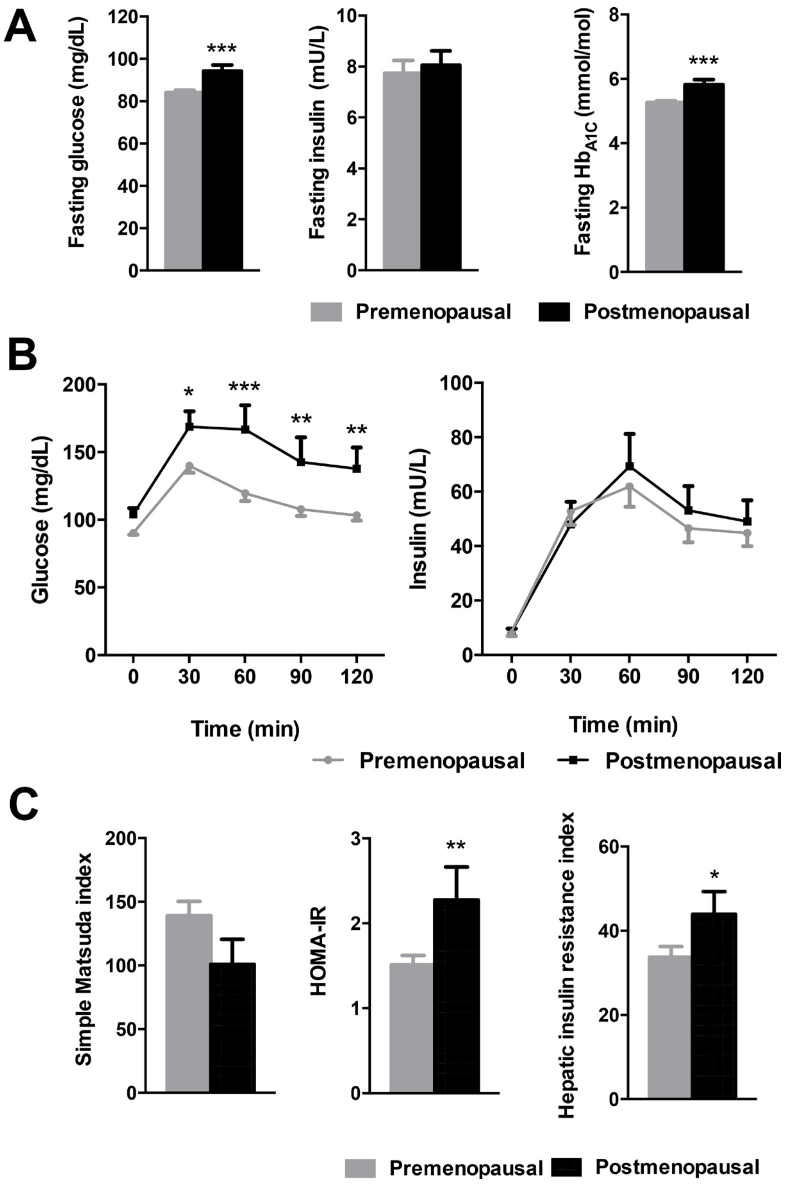

Supplementary Figure 1: Impact of menopausal status on glucose/insulin metabolism parameters in control patients. (**A**) Fasting glucose, insulin and glycohemoglobin levels were determined and analyzed by Student *t*-test (***$p < 0.001$). (**B**) Glucose and insulin levels during the OGTT. Asterisks above each point (*$p < 0.05$; **$p < 0.01$; ***$p < 0.001$) indicate significant differences by Fisher´s LSD test. (**C**) Simple Matsuda index, HOMA-IR and Hepatic insulin resistance index were analyzed by Mann-Whitney U post-hoc test (*$p < 0.05$; **$p < 0.01$). Values represent means ± SEM (premenopause $n = 48$, postmenopause $n = 14$).

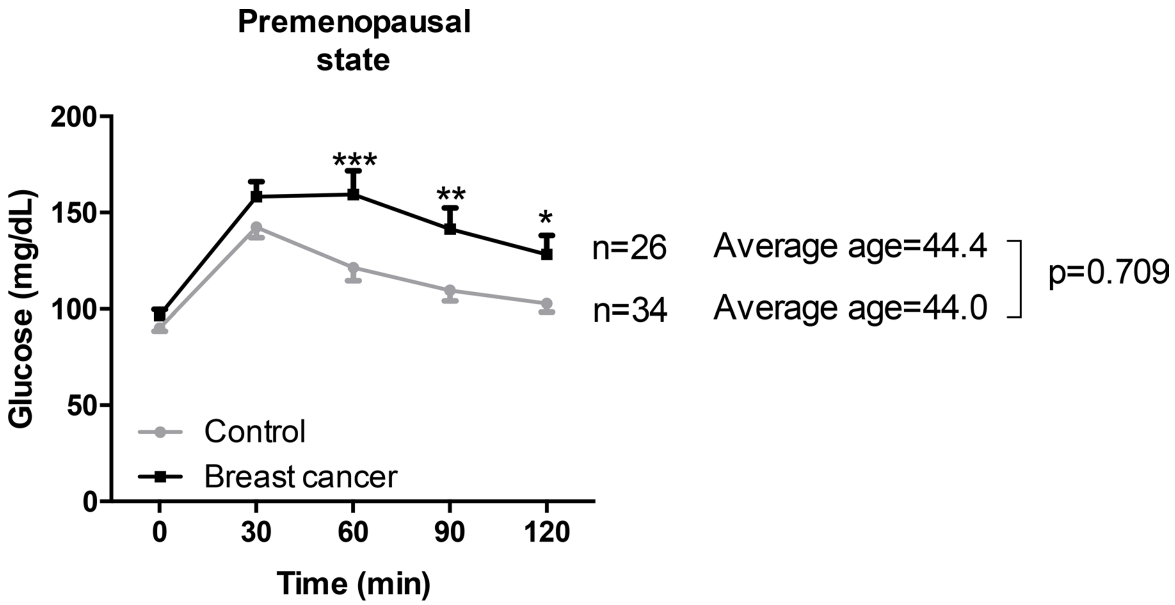

Supplementary Figure 2: Impact of breast cancer presence on glucose levels during the OGTT in a subset of age-matched premenopausal women. Asterisks above each point (*$p < 0.05$; **$p < 0.01$; ***$p < 0.001$) indicate significant differences by Fisher´s LSD test. Values represent means ± SEM (controls $n = 34$, breast cancer patients $n = 26$).

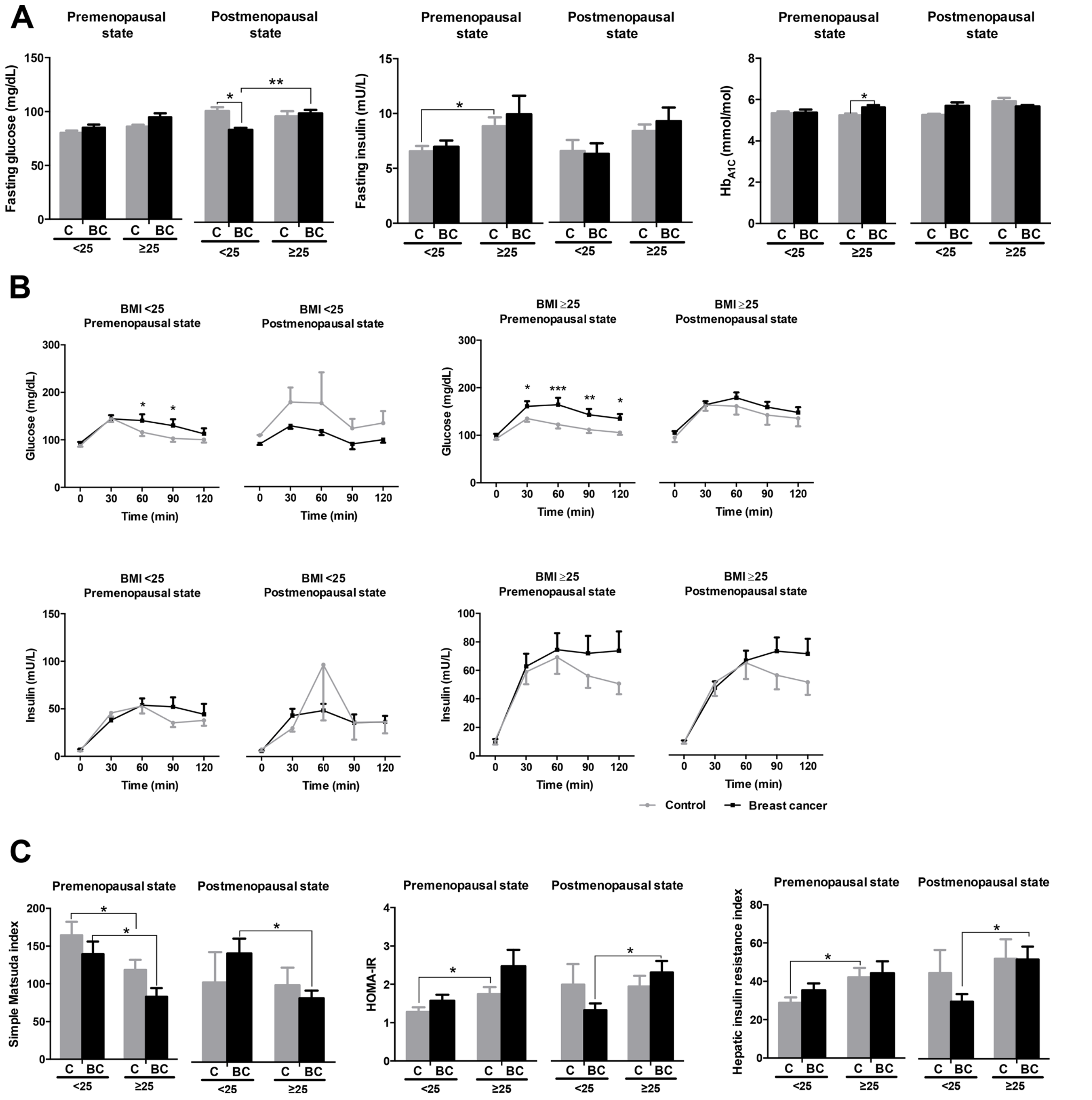

Supplementary Figure 3: Impact of menopausal status on glucose/insulin metabolism parameters in normoweight (BMI < 25) and overweight/obese (BMI ≥ 25) control and breast cancer patients. (**A**) Fasting glucose, insulin and glycohemoglobin levels. (**B**) Glucose and insulin levels during the OGTT. (**C**) Simple Matsuda index, HOMA-IR and Hepatic insulin resistance index. Values represent mean ± SEM of each experimental group (normoweight n = 60; overweight/obese n = 88). Asterisks (*p < 0.05; **p < 0.01; ***p < 0.001) indicate significant differences by Bonferroni/Mann-Whitney U/Fisher´s LSD post-hoc tests. C means control subjects and BC means breast cancer patients.

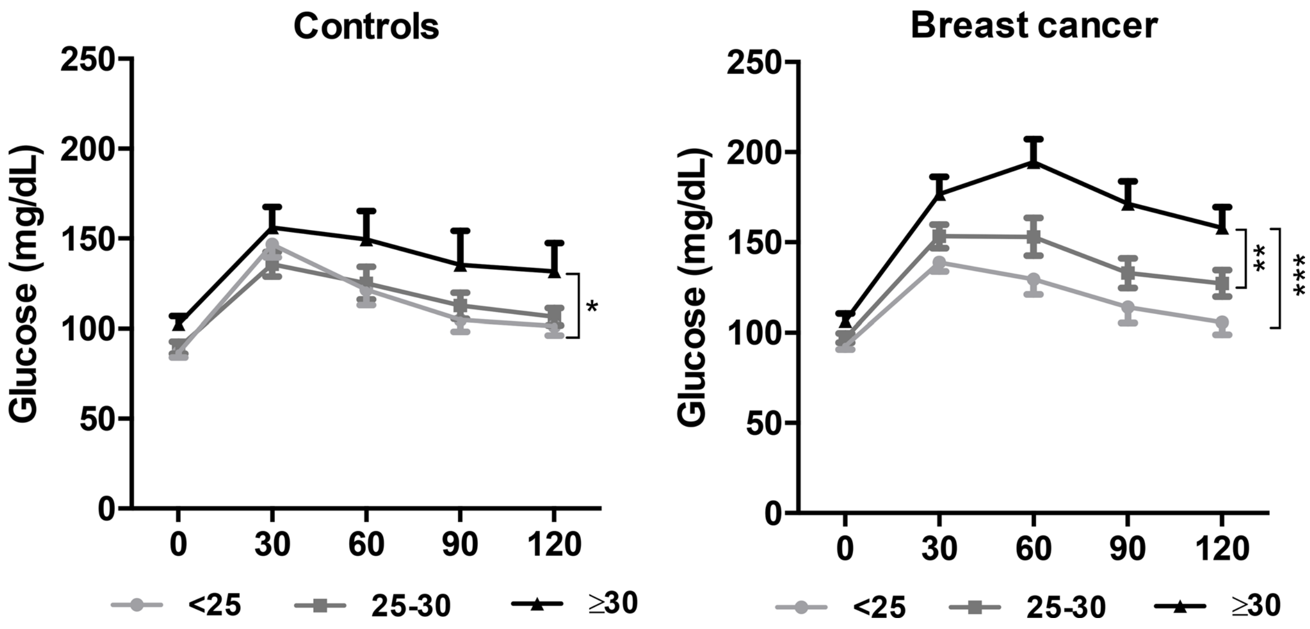

Supplementary Figure 4: Glucose levels during OGTT in normoweight (BMI < 25), overweight (BMI ≥ 25 < 30) and obese (BMI ≥ 30) controls and breast cancer patients. Values represent mean ± SEM of each experimental group (control n = 71; breast cancer n = 77). Asterisks (*p < 0.05; **p < 0.01; ***p < 0.001) indicate significant differences by Fisher´s LSD post-hoc test.

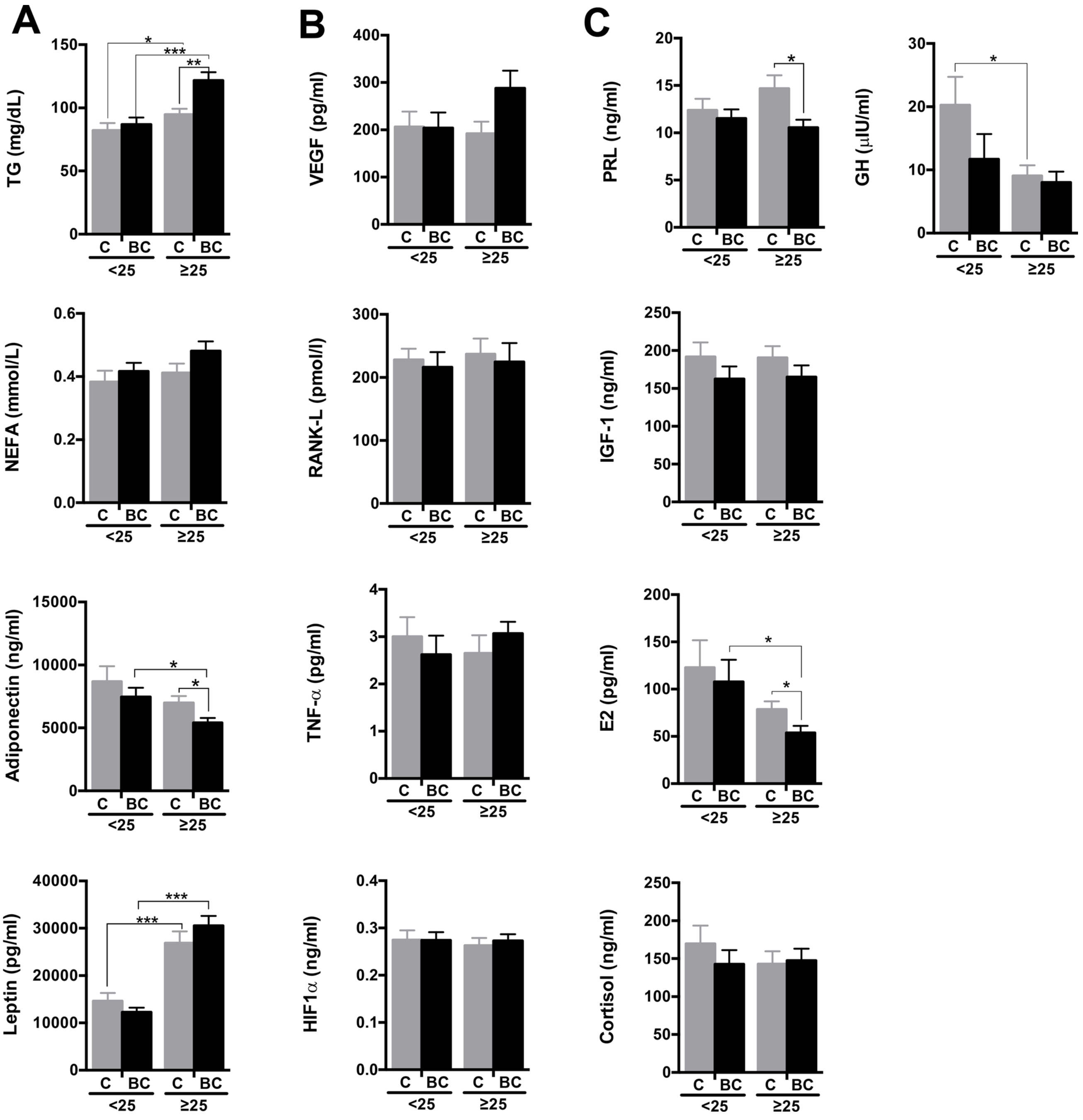

Supplementary Figure 5: Circulating levels of hormones, factors and molecules in normoweight (BMI < 25) and overweight/obese (BMI ≥ 25) control and breast cancer patients. (**A**) Obesity-associated markers (TG, NEFA, adiponectin, leptin), (**B**) tumor-derived factors (VEGF, RANK-L, TNF-α, HIF-1α) and (**C**) other relevant hormones (PRL, IGF-I, estrogens, cortisol, GH) were determined in plasma of cohort individuals. Values represent mean ± SEM of each experimental group (normal weight n = 60; overweight n = 88). Asterisks above bars (*p < 0.05; **p < 0.01; ***p < 0.001) indicate significant differences by Mann-Whitney U post-hoc test. C means control subjects and BC means breast cancer patients.

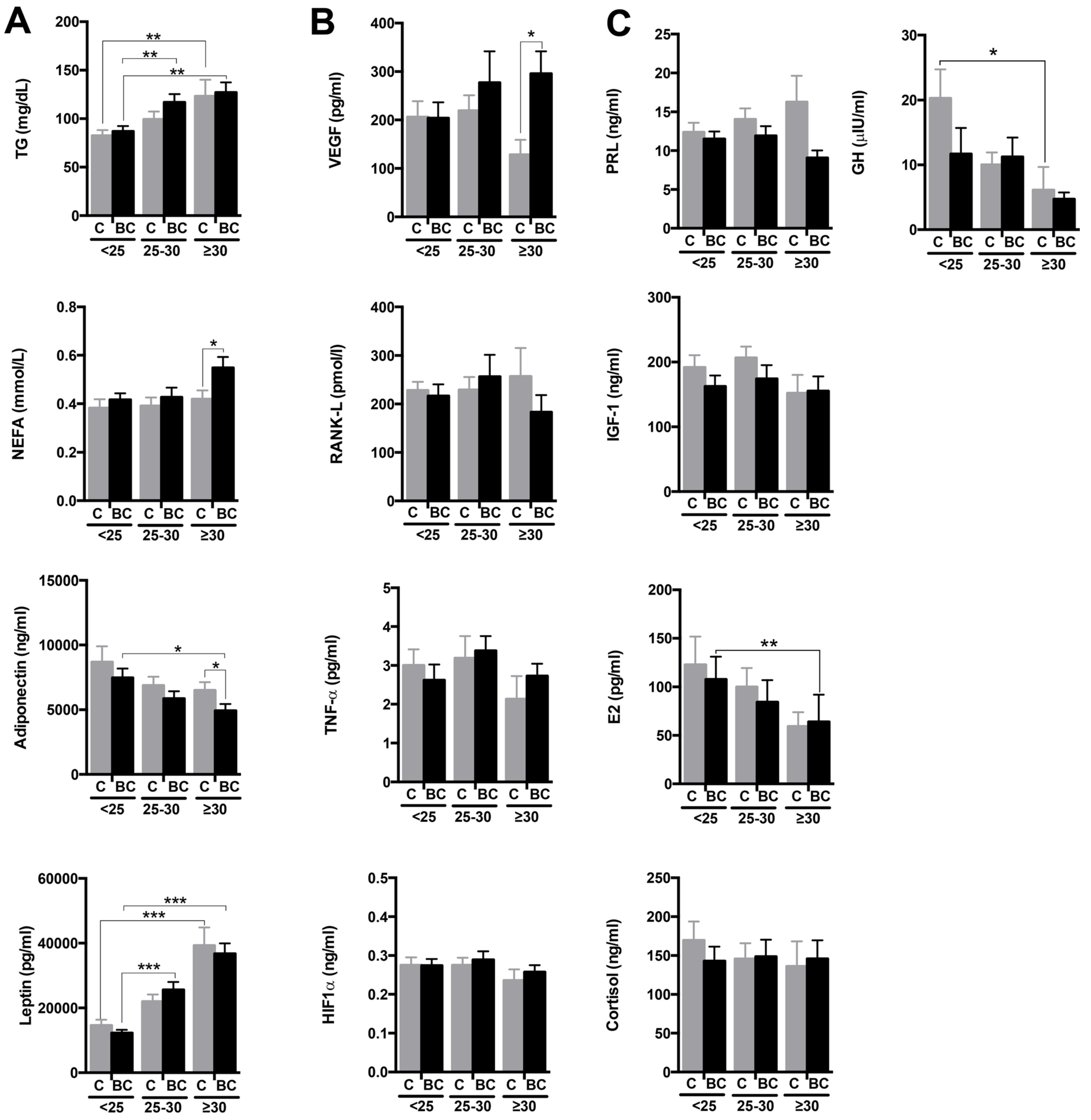

Supplementary Figure 6: Circulating levels of hormones, factors and molecules in normoweight (BMI < 25), overweight (BMI ≥ 25 < 30) and obese (BMI ≥ 30) control and breast cancer patients. (**A**) Obesity-associated markers (TG, NEFA, adiponectin, leptin), (**B**) tumor-derived factors (VEGF, RANK-L, TNF-α, HIF-1α) and (**C**) other relevant hormones (PRL, IGF-I, estrogens, cortisol, GH) were determined in plasma of cohort individuals. Values represent mean ± SEM of each experimental group (normal weight $n = 60$; overweight $n = 54$; obesity $n = 34$). Asterisks above bars (*$p < 0.05$; **$p < 0.01$; ***$p < 0.001$) indicate significant differences by Mann-Whitney U post-hoc test. C means control subjects and BC means breast cancer patients.

Supplementary Table 1: Demographic and clinical parameters of the cohort according to menopausal status

	Control		Breast cancer	
	**Premenopause (*n* = 48)**	**Postmenopause (*n* = 14)**	**Premenopause (*n* = 38)**	**Postmenopause (*n* = 31)**
Age (years)	40.3 ± 1.1	60.2 ± 2.3***	46.3 ± 0.8###	63.1 ± 1.3***
Weight (kg)	67.7 ± 1.7	70.4 ± 2.4	67.8 ± 1.8	70.8 ± 1.8
BMI (kg/m^2)	25.6 ± 0.6	29.5 ± 1.1*	25.5 ± 0.6	29.1 ± 0.8 *
Systolic pressure (mm Hg)	109.3 ± 1.9	128.5 ± 5.1**	118 ± 3.9	136.9 ± 3.6***
Diastolic pressure (mm Hg)	69.1 ± 1.6	77.7 ± 4.6	70.5 ± 2.4	81.1 ± 2.8*
Waist perimeter (cm)	84.1 ± 1.5	96.1 ± 2.6**	85.1 ± 2.1	94.2 ± 2.1**
Number of children	1.9 ± 0.1	2.1 ± 0.2	2.1 ± 0.1	2.6 ± 0.2
Age of menarche	12.2 ± 0.2	12.4 ± 0.3	12.6 ± 0.2	12.8 ± 0.2
Age of first childbirth	26.2 ± 0.8	26.7 ± 1.7	25 ± 0.9	25.1 ± 0.9
Total cholesterol (mg/dL)	196.6 ± 5.2	224.8 ± 5.7*	211.4 ± 7.2	216,1 ± 5.8
HDL cholesterol (mg/dL)	52.7 ± 1.8	54.6 ± 3.4	51.7 ± 1.8	56.9 ± 2.3
LDL cholesterol (mg/dL)	125.3 ± 4.4	142.9 ± 6.0	134.8 ± 5.7	135.6 ± 5.5

*Premenopause vs. Postmenopause

#Prem BCa vs. Prem Control

Values represent mean ± SEM of each parameter in the different groups (premenopause *n* = 86; postmenopause *n* = 45). *P*-value indicate global differences by 2-way ANOVA or Kruskal Wallis tests ($p < 0.05$ was considered significant).

Supplementary Table 2: Influence of age on glucose levels during OGTT

		Breast cancer		Age	
**Glucose levels during OGTT**		**F**	***p* value**	**F**	***p* value**
Control vs. Breast cancer	Without correction	6,111	0,015		
(independent of menopause)	Corrected by age	0,217	0,642	18,905	0,000
Control vs. Breast cancer	Without correction	9,187	0,003		
(Premenopausal state)	Corrected by age	4,142	0,045	2,792	0,099

Differences in glucose levels during OGTT were analyzed with or without correction by age using linear regression analysis. A first group of controls (*n* = 71) and breast cancer patients (*n* = 77) and a second group of premenopausal controls (*n* = 48) and premenopausal breast cancer cases (*n* = 38) were analyzed. F-statistic indicates the variability of the means in each group and p-value indicates statistical significance when is < 0.05.
